# Assessing WildfireGPT: a comparative analysis of AI models for quantitative wildfire spread prediction

**DOI:** 10.1007/s11069-025-07344-7

**Published:** 2025-05-28

**Authors:** Meghana Ramesh, Ziheng Sun, Yunyao Li, Li Zhang, Sai Kiran Annam, Hui Fang, Daniel Tong

**Affiliations:** 1 Center for Spatial Information Science and Systems, Department of Geography and Geoinformation Sciences, Department of Atmospheric Oceanic Earth Sciences, George Mason University, 4087 University Dr STE 3120, Fairfax, VA 22030 USA; 2 Department of Earth and Environmental Sciences, University of Texas, Arlington, USA; 3 CIRES, University of Colorado, Boulder, CO USA; 4 Global Systems Laboratory, Earth System Research Laboratory, NOAA, Boulder, CO USA

**Keywords:** Wildfire prediction, Fire radiative power (FRP), WildfireGPT, TabNet, Machine learning

## Abstract

This study examines the application of WildfireGPT for wildfire forecasting, focusing on its limitations in quantitative predicting Fire Radiative Power (FRP) spread and comparing its performance with a specialized predictive model based on TabNet. While WildfireGPT is widely accessible and convenient for wildfire-related discussions, it lacks the specialized training, real-time data integration, and algorithmic precision required for reliable wildfire forecasting. To highlight these shortcomings, we conducted an experiment using real-world NASA Fire Radiative Power (FRP) datasets. Our TabNet-based model, trained on variables such as Vapor Pressure Deficit (VPD), temperature (T), pressure (P), and Fire Weather Index (FWI), demonstrated high correlation, with low Mean Absolute Error (MAE) and Mean Squared Error (MSE) in forecasting FRP values. In contrast, RAG (retrieval-augmented generation) and LLM (large language model)-based chatbots like WildfireGPT have unreliable performance on quantitative FRP forecasting with the same input data as prompts. The findings underscore the potential risks of over-reliance on general-purpose AI tools like WildfireGPT for quantitative modeling tasks in wildfire management. This study advocates for informed usage of AI tools, emphasizing the necessity of domain-specific models for accurate and actionable wildfire forecasting.

## Introduction

Wildfires have become one of the most significant environmental challenges of our time, posing an immense threat to human lives, properties, and ecosystems (Doerr and Santín 2016). These uncontrolled flames destroy enormous swaths of land every year, resulting in billions of dollars’ worth of losses (U.S. Congress Joint Economic Committee, 2023). There is more to the destruction than just the immediate loss of houses, woods, and infrastructure. The smoke that wildfires emit, which includes dangerous contaminants like lead and fine particulate matter, also has a lasting effect. These airborne pollutants have a long range and can harm people far from the flames by impairing air quality and raising the risk of respiratory and cardiovascular illnesses (Kelly and Fussell 2020).

In response to the growing frequency and severity of wildfires, technology has emerged as a critical tool in wildfire management. LLM-based wildfire-focused chatbots such as WildfireGPT are advanced digital assistants to support prevention, response, and recovery efforts (Xie et al. 2024a, b, c, d; Kaur et al. 2024; Carolan et al. 2024). These tools make wildfire management smarter and faster by helping with decisions, guiding responses, and offering real time insights. These chatbots leverage AI offer a range of functionalities, from real time data analysis and predictive modeling to public communication and educational outreach (Cheng et al. 2024). They also serve as an educational purpose by raising awareness about wildfire safety. They can provide guidance on fire safe practices, such as clearing vegetation around homes, creating defensible spaces, and preparing emergency kits. They not only support emergency teams in making informed decisions but also empower communities to take proactive measures, reducing the overall impact of wildfires.

Among all the work for wildfire management, a key capability in the fight against wildfires is the accurate assessment of Fire Radiative Power (FRP). FRP is a key metric that quantifies the energy emitted as vegetation burns, providing a reliable indication of the fire’s intensity and potential for spread (Kaiser et al. 2012). Understanding FRP is vital for emergency response teams as it allows them to anticipate the behaviour of wildfires, allocate resources effectively, and implement appropriate safety measures. Thus, general damages to the environment are lowered, infrastructure losses reduced, and risks to human life significantly reduced. Predicting Fire Radiative Power (FRP) is a crucial yet challenging task in wildfire management. The process involves analyzing and integrating various environmental factors that significantly influence fire behavior. For instance, the type of fuel, such as the specific vegetation or materials burning, directly impacts the amount of energy a fire will release (Langmann et al. 2009; Keywood et al. 2013; Santín and Doerr 2016).

The TabNet model, identified through an AutoML approach, emerged as a valid baseline for addressing the challenges of FRP prediction. While TabNet’s predictive accuracy underscores its effectiveness, it is important to recognize that a wide array of machine learning models—ranging from SVMs and Random Forests to CNNs and Transformer-based architectures—have already been evaluated for wildfire-related tasks, including FRP estimation (Sun et al. 2024; Wang et al. 2023; Syauqillah et al. 2023; Jain et al. 2020). These studies have established valuable performance benchmarks and model trade-offs for this work.

Although WildfireGPT was not originally developed for direct numerical forecasting tasks, its flexible interface and natural language capabilities may lead users—intentionally or unintentionally—to rely on it for such purposes in real-world wildfire response scenarios. This possibility raises important concerns about reliability, interpretability, and risk. If generative models are used for high-stakes prediction tasks without validation, the consequences could be significant. Therefore, this paper aims to examine WildfireGPT’s performance in quantitative FRP prediction alongside a conventional model like TabNet, in order to assess its suitability, limitations, and potential role in operational wildfire management pipelines.

The rest of this paper is organized as follows: Sect. 2 discusses Data Collection and provides an overview of the dataset and its features. Section 3 covers the WildfireGPT and its comparison model TabNet Model, detailing its architecture and suitability for this task. Section 4 presents Wildfire Prediction results, evaluating the performance of the TabNet model against WildfireGPT. Section 5 summarizes the work and lays out future actions.

## Dataset

The dataset for this research was primarily sourced from the NASA Fire Energetics and Emissions Research platfrom (Davies et al. 2008), which provides detailed MODIS-based fire-related data, including Fire Radiative Power (FRP) values. To ensure the reliability of the analysis, the model was trained using data from the 2020 dataset, and its performance was evaluated on a separate dataset from July 2021. This approach allowed for a robust validation of the model’s predictive accuracy, ensuring that it can generalize well to new, unseen data.

The dataset utilized in this study comprises a comprehensive collection of 61 features and 619,373 rows. These features encompass a wide range of environmental and meteorological parameters for accurately predicting Fire Radiative Power (FRP). The features include weather indices such as the Fire Weather Index (FWI) and Vapor Pressure Deficit (VPD), as well as temperature (T), relative humidity (RH), and wind components (U and V). The dataset includes precipitation (P), rainfall (RAIN), Convective Available Potential Energy (CAPE), soil temperature (ST), and soil moisture (SM). In terms of geographical context, the dataset also provides proximity information through features labelled Nearest_1 to Nearest_24, which indicate the distances to the nearest locations relevant to the data point. Furthermore, vegetation indices such as the Vegetation Condition Index (VCI_AVE), Temperature Condition Index (TCI_AVE), and Vegetation Health Index (VHI_AVE) are included, providing insight into the land’s condition over time. These indices are complemented by total versions (VCI_TOT, TCI_TOT, VHI_TOT) that aggregate the information over broader periods. The dataset also incorporates lagged variables, capturing the state of various parameters up to seven days prior (e.g., FWI_1_days_ago, VPD_1_days_ago, FRP_1_days_ago, etc.). This inclusion of temporal context allows the model to consider the evolution of conditions leading up to each observation, which is crucial for understanding and predicting the dynamic behavior of wildfires.

It is important to note that the value − 999.0 is used throughout the dataset to indicate missing or unavailable data. Handling such missing data appropriately is essential for ensuring that the model’s predictions are based on accurate and complete information. Overall, the diversity and richness of the dataset, with its 61 features and over 619,000 observations, provide a robust foundation for building and training predictive models.

## Methodology

**Fig. 1:**
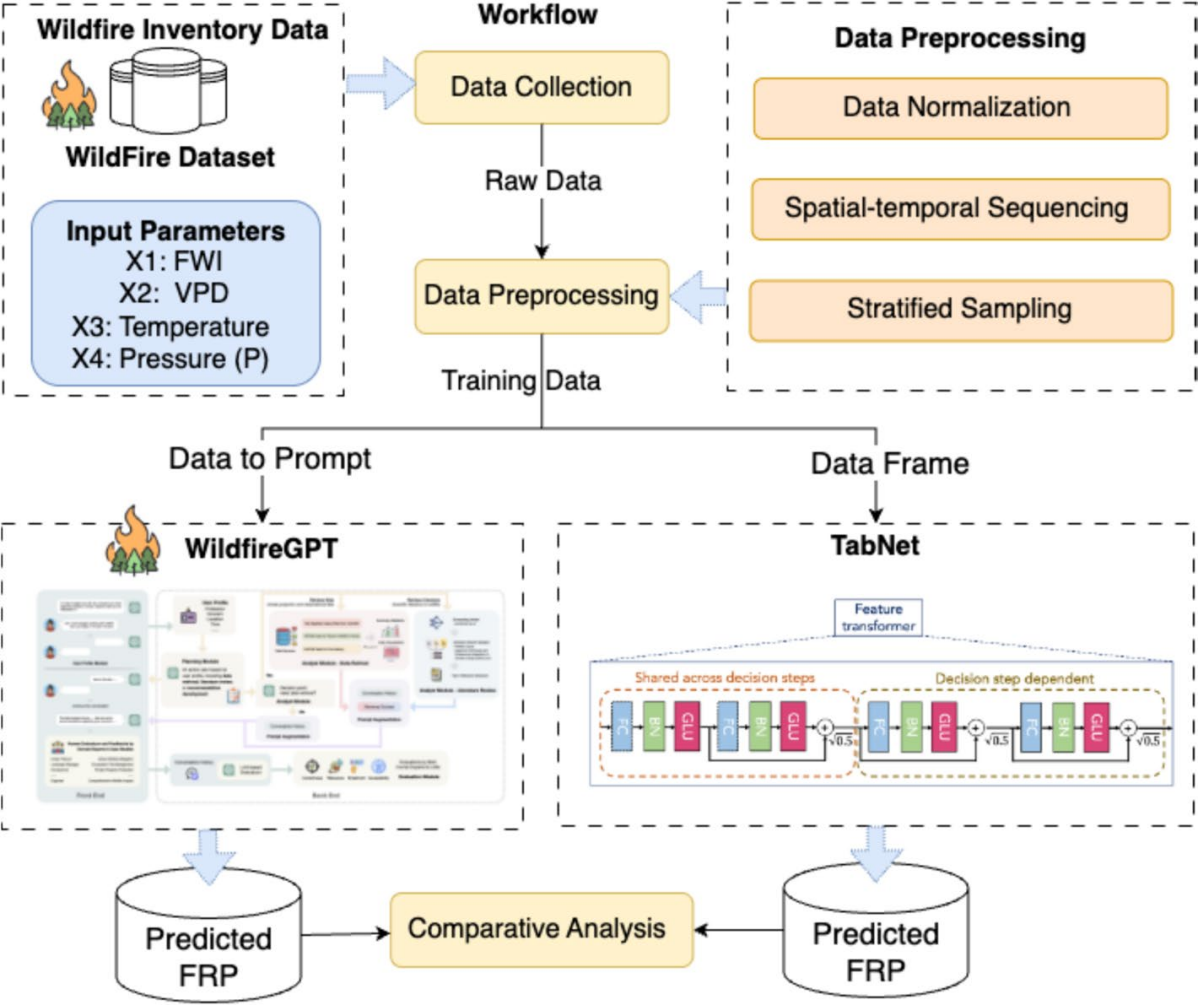
The workflow of comparing WildfireGPT and TabNet

WildfireGPT, despite its versatility as a conversational AI, is often perceived as a tool capable of handling specialized tasks like wildfire forecasting due to the impression brought by its mother model ChatGPT (Biswas 2023). However, this perception can lead to misplaced trust, especially when the model's outputs are used in high stakes scenarios. The tendency to rely on such tools stems from their convenience and the impression that AI can provide definitive answers to complex problems. This section examines WildfireGPT’s capabilities and compares its performance against traditional AI models, such as TabNet, which are specifically designed for predictive tasks. Figure 1 shows our evaluation workflow. The goal is to assess whether WildfireGPT can reliably fulfill precise forecasting needs or if conventional models remain indispensable for accuracy and accountability.

### WildfireGPT

#### What is WildfireGPT

**Fig. 2:**
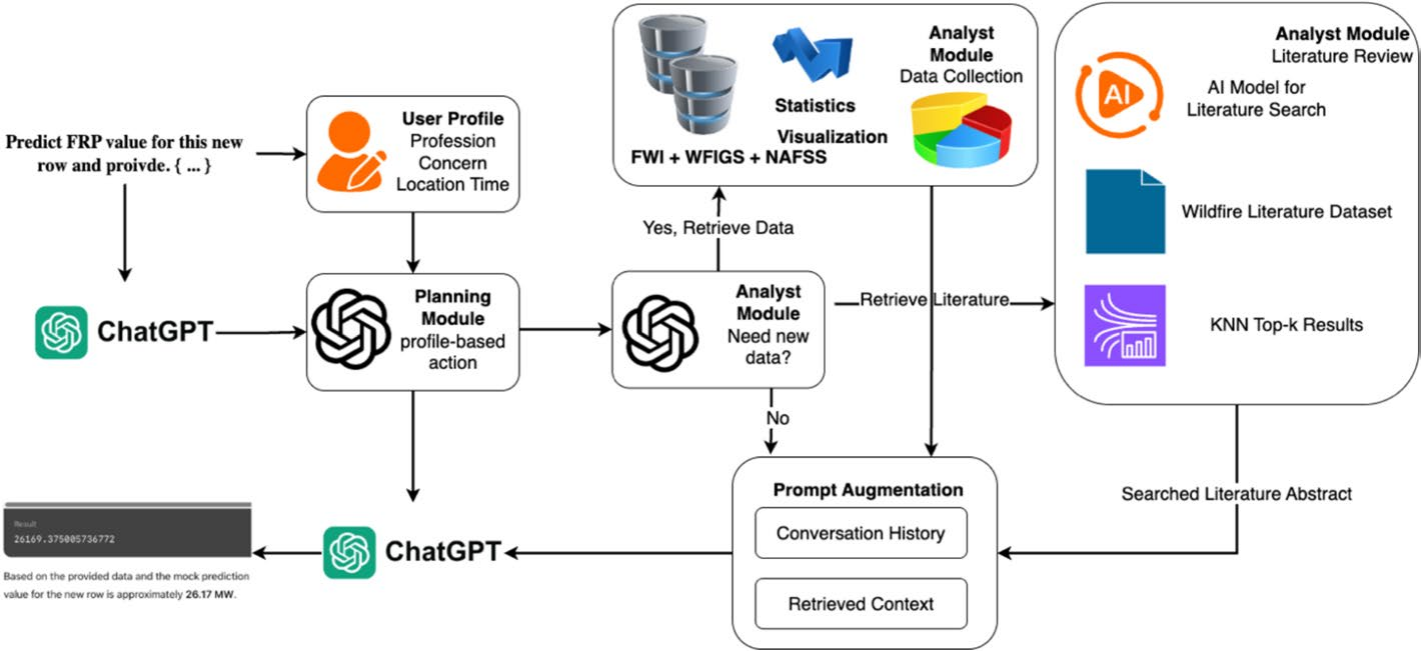
The architecture of WildfireGPT (adapted from Xie et al. 2024a, b, c, d)

WildfireGPT, a derived version of OpenAI’s GPT-4 (Achiam et al. 2023), is a multi-agent retrieval-augmented generation (RAG) system designed to enhance wildfire risk assessment and decision-making through a structured workflow of specialized AI agents (Xie et al. 2024a, b, c, d). The system leverages OpenAI’s Function Calling API to coordinate its agents, including the task orchestrator, user profile assistant, planning assistant, and analyst assistant. The task orchestrator dynamically routes user interactions to the appropriate agent, ensuring seamless task execution. Each assistant is designed with prompt engineering techniques to guide interactions, gather user-specific information, and generate tailored wildfire risk insights. To ensure continuity, the system incorporates a conversation-resuming mechanism that preserves thread history, allowing users to return to previous discussions effortlessly (as shown in Fig. 2).

For data-driven analysis, WildfireGPT integrates multiple geospatial and environmental datasets, including Fire Weather Index (FWI) projections, historical wildfire incident records, and paleofire data. The system employs geospatial data processing techniques, such as GeoJsonLayer rendering for visualization and spatial mapping algorithms to associate user-defined locations with corresponding datasets. Census data is also incorporated to provide socio-economic risk assessments, adding depth to wildfire impact evaluations. WildfireGPT’s literature retrieval capabilities utilize a FAISS vector store (Johnson et al. 2019) and SentenceTransformers embeddings (Reimers et al. 2019) to identify relevant research papers, ensuring that recommendations are informed by scientific literature. DOI validation through CrossRef metadata ensures data integrity and credibility. The analyst assistant synthesizes wildfire risk assessments by retrieving, visualizing, and interpreting data, generating interactive maps, structured reports, and tailored recommendations. The system classifies FWI data into six risk categories using the Canadian Forest Fire Weather Index framework and presents both historical and future fire trends across seasons. The retrieval process is designed for efficiency, employing K-nearest neighbor (KNN) searches in the FAISS vector database for literature recommendations. The entire architecture emphasizes modularity and scalability, allowing for future expansions in datasets, risk modeling techniques, and real-time wildfire monitoring applications.

WildfireGPT serves as a resource for fire scientists, land managers, and wildfire management professionals, offering them a tool to enhance their understanding and decision-making processes. It is equipped to handle technical queries that require a deep understanding of fire science, making it an invaluable tool for professionals seeking to refine their strategies for fire prevention, suppression, and ecological restoration. While it offers a wealth of knowledge and insight, it is designed to complement, not replace, professional judgment, particularly in agent, real-time decision-making scenarios. By providing detailed, science-based responses, WildfireGPT empowers users to apply the most current and relevant knowledge to their specific contexts, ultimately supporting more effective wildfire management and mitigation efforts. 

#### Sample prompts for WildfireGPT

As shown in Table 1, to construct a prompt for WildfireGPT, we flatten all the columns from our training data and merge them into one coherent sentence. Each prompt contains key environmental variables such as the Fire Weather Index (FWI), Vapor Pressure Deficit (VPD), temperature, relative humidity, wind components, atmospheric pressure, rainfall, CAPE, soil temperature, soil moisture, and other relevant historical FRP and meteorological data. This combined information is then presented as a single sentence where we ask WildfireGPT to forecast one exact FRP value. The prompt is designed to provide a clear and concise input to forecast the FRP, based on the environmental conditions provided.

**Table 1:** Example prompts to WildfireGPT

Example Prompts	Answers
Now predict FRP given: FWI is 9.0, VPD is 0.683594, temperature is 301.15, relative humidity is 298.64, U-wind is 0.362334, V-wind is 0.523227, pressure is 101,000, rainfall is 44.15, CAPE is 0, soil temperature is 295.0, soil moisture is 0.20, nearest neighbors 1–24 range from 0.0 to 5.0, land use is shrubland, average VCI is 45, average TCI is 50, average VHI is 55, total VCI is 40, total TCI is 41, total VHI is 45, FWI 1-day ago is 9.0, FWI 2-days ago is 9.0, VPD 1-day ago is 0.646, VPD 2-days ago is 0.65, temperature 1-day ago is 302.0, temperature 2-days ago is 300.0, rainfall 1-day ago is 45.47, rainfall 2-days ago is 40.85	Predicted FRP = 325w/m2 (true FRP = 6.1 w/m2)
Now predict FRP given: FWI is 48.23, VPD is 1.2, temperature is 300.25, relative humidity is 298.25, U-wind is -0.0867, V-wind is 1.3965, pressure is 101,200, rainfall is 12.03, CAPE is 0, soil temperature is 297.0, soil moisture is 0.15, nearest neighbors 1–24 range from 0.0 to 3.0, land use is mixed forest, average VCI is 48, average TCI is 50, average VHI is 50, total VCI is 47, total TCI is 46, total VHI is 45, FWI 1-day ago is 48.23, FWI 2-days ago is 47.18, VPD 1-day ago is 1.25, VPD 2-days ago is 1.3, temperature 1-day ago is 301.1, temperature 2-days ago is 299.5, rainfall 1-day ago is 7.57, rainfall 2-days ago is 13.3	Predicted FRP = 750 w/m2 (true FRP = 37.22 w/m2)
Now predict FRP given: FWI is 44.36, VPD is 1.2, temperature is 293.33, relative humidity is 292.1, U-wind is -0.7042, V-wind is 2.014, pressure is 101,500, rainfall is 6.48, CAPE is 0, soil temperature is 295.0, soil moisture is 0.18, nearest neighbors 1–24 range from 0.0 to 3.0, land use is grassland, average VCI is 69, average TCI is 68, average VHI is 70, total VCI is 65, total TCI is 68, total VHI is 70, FWI 1-day ago is 44.36, FWI 2-days ago is 43.15, VPD 1-day ago is 1.5, VPD 2-days ago is 1.4, temperature 1-day ago is 294.0, temperature 2-days ago is 292.5, rainfall 1-day ago is 6.87, rainfall 2-days ago is 9.0	Predicted FRP = 650 w/m2 (true FRP = 6.77 w/m2)

#### Known limitation of WildfireGPT

Despite not being specifically designed for quantitative wildfire forecasting, WildfireGPT is often used for this purpose due to its accessibility, ease of use, and ability to quickly provide information in a conversational format. Users turn to WildfireGPT when they need immediate insights or guidance, as it can synthesize general knowledge and provide actionable recommendations based on user input. Its ability to interpret complex queries and present responses in a simplified manner makes it an appealing tool for those without access to specialized forecasting software. As mentioned in the original papers (Xie et al. 2024a, b, c, d), this reliance highlights the growing need for accessible AI tools, even in scenarios where they might not meet all technical requirements for specialized applications like wildfire forecasting.

One significant theoretical limitation of WildfireGPT lies in its reliance on RAG to combine the latest data and literature search results into its responses. While RAG enables WildfireGPT to fetch relevant information from external sources, it still faces challenges in integrating and synthesizing this data in real-time to provide accurate, actionable predictions (Laban et al. 2024; Jin et al. 2024). The model's capacity to forecast specific wildfire behavior quantitatively is inherently limited by its reliance on general language processing rather than specialized predictive models. This reliance on retrieved data means that WildfireGPT’s forecasts might not always reflect the complex, dynamic interactions between environmental variables such as wind, temperature, humidity, and vegetation type, which are crucial in wildfire prediction (Coen et al. 2013). As a result, while RAG can enhance the model's responses with up-to-date information, its inability to model physical dynamics directly hampers its accuracy in providing precise numerical predictions (Cherian et al. 2024).

Although RAG helps WildfireGPT to access relevant external literature, the model's performance is restricted by the quality and timeliness of the literature that are retrieved based on simple KNN clustering, and it may fail to account for local, on-the-ground factors such as ignition points or immediate weather shifts for in-situ tasks. Consequently, the model's forecasts can become outdated or overly generalized, reducing their effectiveness in operational wildfire management, where time-sensitive and geographically specific predictions are critical (Fan et al. 2024).

Despite these challenges, many general users may still turn to WildfireGPT for quantitative wildfire forecasting and place undue trust in its results, potentially leading to catastrophic consequences. Therefore, we conducted this research to evaluate its reliability and provide guidance on its appropriate use.

### Conventional TabNet model

#### Performance metric calculation

To compare the TabNet and WildfireGPT model performance, we will use the common regression metrics: Mean Squared Error (MSE), Mean Absolute Error (MAE), R2 score, and Mean Absolute Percentage Error (MAPE). Their equations are listed below. The MSE gives more weight to larger errors.

$$MSE = \frac{1}{n}\sum\limits_{i = 1}^{n} {(y_{i} - \hat{y}_{i} )}$$

The MAE measures the average magnitude of errors in the predictions.

$$MAE = \frac{1}{n}\sum\limits_{i = 1}^{n} {\left| {y_{i} - \hat{y}_{i} } \right|}$$

The R2 score reflects the proportion of variance in the actual values that is predictable from the predicted values.

$$R^{2} = \frac{{\sum\nolimits_{i = 1}^{n} {(y_{i} - \hat{y}_{i} )^{2} } }}{{\sum\nolimits_{i = 1}^{n} {(y_{i} - \overline{y}_{i} )^{2} } }}$$

Finally, the MAPE offers insight into the percentage error of the predictions.

$$MAPE = \frac{1}{n}\sum\limits_{i = 1}^{n} {\left| {\frac{{y_{i} - \hat{y}_{i} }}{{y_{i} }}} \right|} \times 100$$

The ${y}_{i}$ is the predicted FRP, $\widehat{{y}_{i}}$ is the true FRP, $\overline{y }$ is the mean of actual FRP values, n is the number of FRP predictions. These metrics collectively provide a comprehensive understanding of the model’s predictive accuracy and reliability.

#### AI model selection and evaluation

We use a subset of the training data (74,550 rows) to test several well-known AI models, including TabNet, Random Forest, XGBoost, and LightGBM. TabNet is a deep learning model specifically designed for tabular data, utilizing a sequential attention mechanism that selects features at each decision step, which improves both interpretability and predictive performance (Arik and Pfister 2021). Random Forest is an ensemble method based on decision trees (Breiman 2001). XGBoost uses gradient boosting to optimize the loss function iteratively (Chen and Guestrin 2016). LightGBM, another gradient boosting framework, has faster training speed and requires lower memory usage compared to other gradient boosting algorithms (Ke et al. 2017). We used the Optuna library for hyperparameter optimization. Optuna is an open-source tool designed to automate the process of hyperparameter tuning by exploring the hyperparameter space efficiently (Akiba et al. 2019). In our study, we defined an objective function that trains each model and evaluates its performance using Root Mean Squared Error (RMSE) on the validation set. Optuna’s Bayesian optimization approach allowed us to search the hyperparameter space more intelligently than random or grid search methods. We ran five trials on each model. To assess each trial, we relied on RMSE and MAE that measures the average magnitude of errors between the predicted and actual values. For each trial, we trained the models using a training dataset and evaluated their performance on a separate validation dataset to ensure unbiased performance assessment. To prevent overfitting, where the model becomes too specialized in the training data and performs poorly on unseen data, we implemented early stopping. Early stopping is a regularization technique that halts the training process if the model’s performance on the validation set does not improve after a specified number of iterations (Prechelt 2002). This selection approach ensures that the model performs well on new, unseen data, for reliable predictions in real world wildfire scenarios. Based on our results (shown in Table 2), TabNet has the lowest RMSE and therefore is chosen as our final model to compare with WildfireGPT.

**Table 2:** Preliminary comparison of non-LLM AI models

Model	Optuna Hyperparameters	RMSE	MAE
Random Forest	n_estimators, max_depth, min_samples_split	15.9844	10.6483
XGBoost	n_estimators, max_depth, learning_rate, subsample	16.5935	10.6868
LightGBM	n_estimators, max_depth, learning_rate, num_leaves	16.0797	10.5646
TabNet (50 epoch)	n_d, n_a, n_steps, gamma	15.9555	10.5568

#### Analyzing features

After identifying TabNet as the best model, the next step is analyzing feature importance. This analysis is fundamental to understanding which features are driving the model’s decisions and which ones may be contributing less significantly. The process begins with the feature importance analysis, which is followed by the elimination of columns that are deemed not highly important. This step focuses on analyzing the significance of each feature in the dataset. The data, which is stored in a CSV file, is loaded into a DataFrame, and the feature matrix is prepared by excluding the target variable, FRP. This matrix is then passed to the TabNet model to generate predictions. The act of running the data through the model sets the stage for examining how different features contribute to the model’s output.

The feature importance analysis is performed using the explainable method provided by the TabNet model. This method produces a matrix that reveals the importance of each feature across all the decision-making steps within the model. By averaging the importance scores across all samples, the code calculates a global importance score for each feature. Based on the analysis of the TabNet model, several features contributed little to no significance in predicting the target variable. So, these low importance features were systematically removed from the dataset, to streamline the model and focus on the most relevant data. Specifically, the columns FWI, VPD, HT, and T were among those found to have no significant impact. Lagged variables, such as FWI_1_days_ago, VPD_1_days_ago, and P_1_days_ago, along with similar lagged features for multiple days (2_days_ago through 7_days_ago), were also removed. These lagged features, which recorded past weather and fire radiative power (FRP) values, were determined to be redundant or irrelevant for the model’s current structure.

The spatial neighbor related features such as Nearest_3, Nearest_4, and Nearest_5, as well as historical FRP data (FRP_1_days_ago through FRP_7_days_ago), were excluded from the dataset. The removal reduces the dimensionality , potentially improving the model’s performance by eliminating noise and preventing overfitting to irrelevant features. It ensures that the final model is not only simpler and more computationally efficient but also more focused on the variables that truly matter.

#### Model training

The model training process revolves around leveraging the TabNet Regressor to accurately predict Fire Radiative Power (FRP). The input features (X) are separated from the target variable (y). The input features are those with non-zero feature importance features, while the target variable represents the FRP values. The TabNet model is then configured with a set of hyperparameters that have been specifically chosen to optimize its performance including the width of the decision layers (n_d) and attention layers (n_a), to capture complex patterns in the data. The number of decision steps (n_steps) and regularization parameters (gamma and lambda_sparse) are tuned to balance the model’s expressiveness with its ability to avoid overfitting. Aanother key step is the implementation of k-fold cross validation to enhance the model’s robustness by dividing the dataset into multiple subsets (folds). In each iteration, the model is trained on a portion of the data (the training set) and validated on a different portion (the validation set). By rotating the validation set across different folds, the model is tested across various data configurations, reducing the likelihood of overfitting and ensuring that the model’s performance is consistent across different data splits.

During each fold, the model is trained using the Adam optimizer (Zhang 2018), an optimization algorithm known for its efficiency in handling complex models like TabNet. The learning process is further refined through the application of early stopping criteria, which monitor the model’s performance on the validation set. If the validation performance does not improve over a certain number of epochs, the training process is halted to prevent overfitting. These metrics provide a comprehensive assessment of the model’s accuracy, its ability to minimize errors, and its overall fit to the data. The model with the best performance metrics across all folds is selected as the final model.

## Results and evaluation

**Fig. 3:**
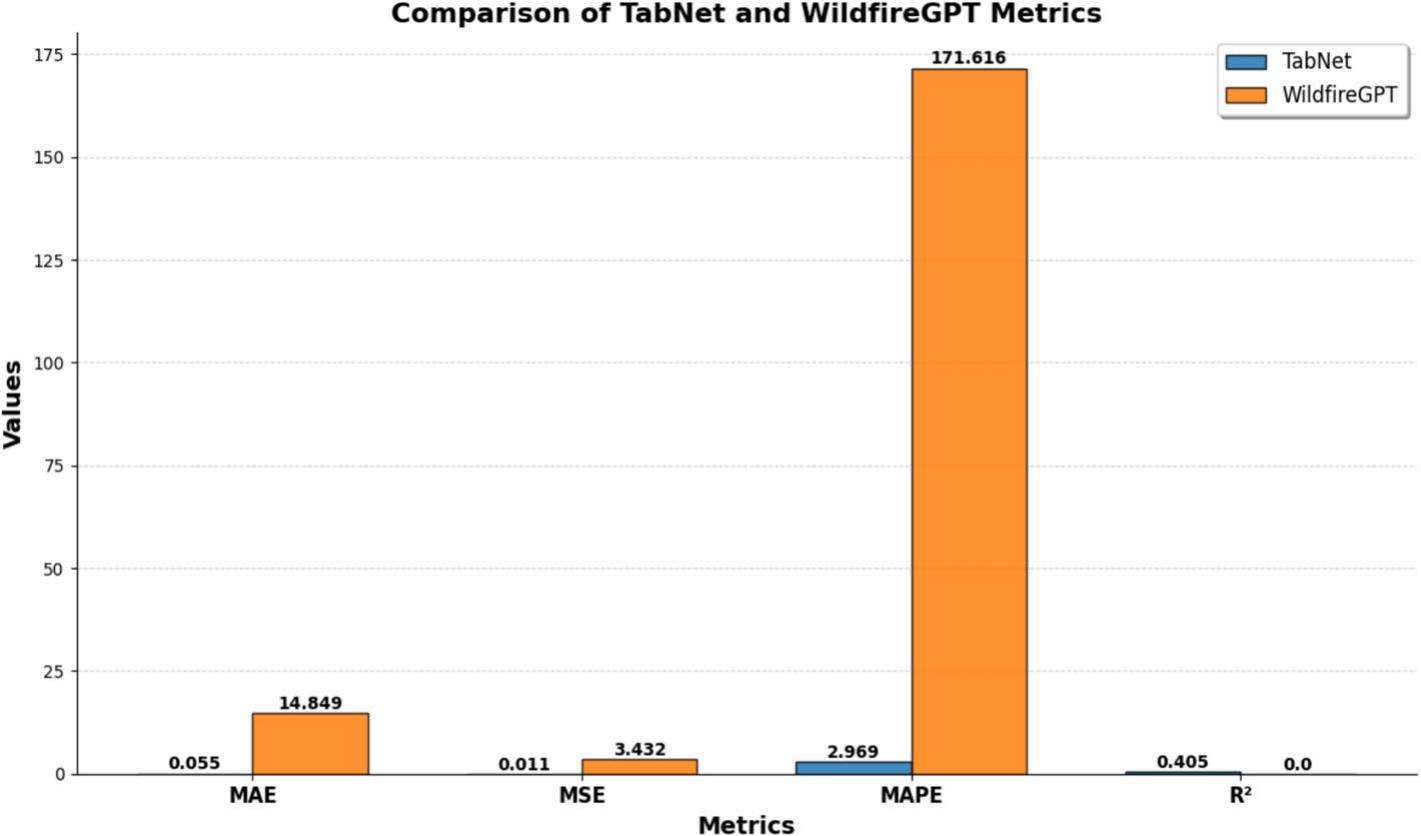
Comparison of Tabnet and WildfireGPT results

This section will compare TabNet and WildfireGPT and reveal the differences across all the metrics, highlighting the strengths and weaknesses. As shown in Fig. 3, the results show that TabNet significantly outperforms WildfireGPT, making it a more reliable and accurate model for predicting FRP.

As shown in Fig. 3, TabNet has impressive accuracy, with a Mean Absolute Error (MAE) of 0.055. This small MAE means that TabNet’s predictions are very close to the real FRP values. On the other hand, WildfireGPT has a much bigger MAE of 14.849, which points to a big gap from the actual FRP values. It means a basic flaw in WildfireGPT’s ability to predict FRP. The big difference suggests that while TabNet does a good job at keeping prediction errors small, WildfireGPT has trouble reaching the same level of accuracy. Mean Squared Error (MSE) analysis shows that TabNet maintains consistency in its predictions. TabNet’s MSE is 0.011, indicating that the variance in its prediction errors is quite low. It means that the model’s errors are tightly clustered around the true values, which is an important characteristic for ensuring reliability in model predictions. WildfireGPT, on the other hand, records an MSE of 3.432, which is significantly higher than that of TabNet. It indicates greater variability in its prediction errors, meaning that its predictions are neither accurate nor consistent. The substantial gap in MSE further underscores TabNet’s robustness and WildfireGPT's unreliability .

The Mean Absolute Percentage Error (MAPE) measures the error as a percentage of the real values. TabNet has a very low MAPE of 2.969%, which shows its errors are small compared to the size of the FRP values. This low percentage error is particularly important in wildfire prediction, where the magnitude of the FRP values can vary widely. A model with a low MAPE, like TabNet, can handle these changes better giving good predictions in different situations. On the other hand, WildfireGPT has a MAPE of 171.616%, which is way too high and means its predictions are often way off compared to the real values. It suggests that WildfireGPT’s predictions are not just wrong in absolute terms, but also fail to stay accurate for different magnitudes of FRP values. Such a high MAPE can severely undermine the utility of WildfireGPT in operational settings.

The coefficient of determination (R2) measures how well the model explains the variability in the FRP data. TabNet achieves an R2 value of 0.405, that means it can explain approximately 40.5% of the variance in the FRP values. This shows TabNet has some predictive ability, which is way better than WildfireGPT’s R2 value of 0. A zero R2 value means WildfireGPT can’t find any real links between the input features and the FRP values so its predictions are no better than guessing. TabNet can explain a good chunk of the data’s variance, which proves it works well as a predictive model. However, WildfireGPT predictions are similar or even worse than random guessing or just using the average history FRP. 

These findings tell us TabNet is a trustworthy and precise model for predicting wildfire related numbers FRP. It has low error rates across several metrics and can explain a fair amount of the data. This makes it a better fit for jobs that need exact and steady wildfire predictions. On the other hand, WildfireGPT’s performance shows it’s not great for these quantitative prediction tasks. Its high error rates and inability to explain the data’s variance suggest it’s not ready to directly model wildfires.

## Conclusion and future work

While WildfireGPT is a valuable tool for answering knowledge-based queries and providing general insights, it is not suitable for quantitative forecasting tasks such as predicting Fire Radiative Power (FRP). It cannot provide accurate, reliable quantitative predictions for real-world wildfire events. As a result, it is recommended that users avoid relying on WildfireGPT for quantitative forecasting tasks. Conventional AI models designed for predictive tasks, such as those tailored specifically for wildfire behavior, should be used in real-world cases to ensure more accurate and actionable outcomes.

 In future, the potential to rethink the integration of RAG and conventional AI models exists, and there may be opportunities to combine the strengths of both approaches in future versions. Such hybrid models could leverage WildfireGPT’s ability to gather and synthesize knowledge from large datasets alongside specialized forecasting models for more accurate and timely predictions. However, until such advancements are made, users should prioritize the use of proven, domain-specific models for quantitative wildfire prediction tasks, ensuring that forecasting tools are both reliable and effective in managing wildfire risks.
